# Ambulatory induction phase treatment of cryptococcal meningitis in HIV integrated primary care clinics, Yangon, Myanmar

**DOI:** 10.1186/s12879-021-06049-z

**Published:** 2021-04-21

**Authors:** Clare E. Warrell, Catriona Macrae, Alistair R. D. McLean, Edmund Wilkins, Elizabeth A. Ashley, Frank Smithuis, Ni Ni Tun

**Affiliations:** 1 Myanmar Oxford Clinical Research Unit (MOCRU), Yangon, Myanmar; 2 Medical Action Myanmar, Yangon, Myanmar; 3 Centre for Tropical Medicine and Global Health, Nuffield Department of Clinical Medicine, University of Oxford, Oxford, OX3 7FZ UK; 4 Lao-Oxford-Mahosot Hospital-Wellcome Trust Research Unit, Microbiology Laboratory, Mahosot Hospital, Vientiane, Lao People’s Democratic Republic

**Keywords:** Cryptococcal meningitis, HIV, Opportunistic infections

## Abstract

**Background:**

Cryptococcal meningitis (CM) is a common HIV-associated opportunistic-infection worldwide. Existing literature focusses on hospital-based outcomes of induction treatment. This paper reviews outpatient management in integrated primary care clinics in Yangon.

**Method:**

This retrospective case note review analyses a Myanmar HIV-positive patient cohort managed using ambulatory induction-phase treatment with intravenous amphotericin-B-deoxycholate (0.7–1.0 mg/kg) and oral fluconazole (800 mg orally/day).

**Results:**

Seventy-six patients were diagnosed between 2010 and 2017. The median age of patients diagnosed was 35 years, 63% were male and 33 (45%) were on concurrent treatment for tuberculosis. The median CD4 count was 60 at the time of diagnosis. Amphotericin-B-deoxycholate infusions precipitated 56 episodes of toxicity, namely hypokalaemia, nephrotoxicity, anaemia, febrile reactions, phlebitis, observed in 44 patients (58%). One-year survival (86%) was higher than existing hospital-based treatment studies.

**Conclusion:**

Ambulation of patients in this cohort saved 1029 hospital bed days and had better survival outcomes when compared to hospital-based studies in other resource constrained settings.

## Background

Cryptococcal meningitis is responsible for an estimated 15% of AIDS related deaths globally. In 2014 there were thought to be 223,100 cases in people living with HIV [1]. In Myanmar, HIV prevalence in adults is 0.57% [2]. It is an epidemic concentrated in key affected populations (KAPs), including female sex workers, men who have sex with men and people who inject drugs, who are generally reluctant to utilize hospital care, for fear of prejudice.

Until recently the World Health Organization (WHO) treatment guidelines advised a 14-day course of amphotericin-B-deoxycholate based treatment for cryptococcal meningitis. Current (2018) WHO recommendations promote one-week dual therapy with amphotericin-B-deoxycholate (1.0 mg/kg/day) and flucytosine (100 mg/kg/ day, divided into four doses per day) for induction phase treatment, followed by one week of fluconazole (1200 mg/day for adults). Alternative induction phase regimens recommended depending on drug availability are; two weeks of fluconazole (1200 mg daily for adults) and flucytosine (100 mg/kg/day, divided into four doses per day) or two weeks of amphotericin- B- deoxycholate (1.0 mg/kg/day) and fluconazole (1200 mg daily for adults) [3]. However, flucytosine is prohibitively expensive for most low and middle income countries (LMICs), and is usually not available [4]; therefore amphotericin-B-deoxycholate and high dose fluconazole are still used in these settings. These drugs are not without risk. Complications associated with amphotericin-B-deoxycholate include nephrotoxicity, hypokalaemia, anaemia, phlebitis and febrile reactions, hence close monitoring is required [5–7].

Ambulatory management for cryptococcal meningitis does not feature in existing national or international guidance. Outpatient amphotericin-B-deoxycholate infusion therapy has historically been used for visceral leishmaniasis treatment [8]. Published literature concentrates on hospital-based mortality outcomes for confirmed cryptococcal meningitis cases. However, in resource-constrained settings outpatient management may be the only option available to deliver life-saving treatment due to social and financial barriers to hospital-based care [9].

Medical Action Myanmar (MAM) is a medical aid organisation providing outpatient HIV care integrated with other services (primary care, tuberculosis, sexual health, antenatal care) free of charge in the most socially deprived areas of the country. The MAM HIV satellite site model-of-care has been described elsewhere [9]. These clinics have been providing prevention and treatment of opportunistic infections since 2009. HIV care is prioritised in KAPs, the most vulnerable and stigmatized groups in society. The ambulatory approach to treating cryptococcal meningitis was a compromise to ensure patients got the treatment they needed within the day-time clinic hours, because many patients were unwilling to be admitted to hospital.

This brief report describes outcomes of HIV positive patients diagnosed with cryptococcal meningitis and treated in an ambulatory setting.

## Methods

A retrospective case note review was carried out of all cryptococcal meningitis patients presenting to four outpatient clinics (9 am-5 pm, 7 days per week) from 2010 to 2017 in Yangon’s most deprived townships. Patient notes were identified from the variables “cryptococcosis extrapulmonary (e.g. meningitis)” and “mycosis disseminated” in the clinic database (FUCHIA; Epicentre). Patients included had a compatible clinical history and met the following criteria: cryptococci seen on CSF microscopy (India Ink), blood or CSF cryptococcal antigen positive, or split skin smear (India ink stain) microscopy positive. Case notes across four MAM clinics were reviewed to extract information including demographic details, presenting symptoms, full blood count, serum creatinine (Cr) and electrolytes, observations at the time of cryptococcal diagnosis, amphotericin treatment regimen, management, complications and outcome (death, admission to hospital, lost to follow up, survival at 1 year.)

Patients were managed on an ambulatory basis as follows; intravenous amphotericin B-deoxycholate (0.7–1.0 mg/kg) plus fluconazole (800 mg orally/day) for at least 2 weeks, followed by a consolidation phase with fluconazole (800 mg orally/day) for 8 weeks followed by maintenance fluconazole (200 mg orally/day) until a sustained CD4 count above 200/mm^3^. Before each amphotericin infusion patients were pre-hydrated with normal saline and given 2 tablets of potassium chloride (Slow-K, 600 mg) twice daily to minimise risk of toxicity.

Diagnostic procedures, monitoring and treatment were carried out during clinic opening hours. These included twice weekly blood tests (sent to external laboratories) and repeat lumbar punctures. Patients were sent home at night with an information leaflet for emergency referral to hospital if something happened at home. Emergency taxi fares were provided in advance to all the patients.

Treatment complications used by clinicians reviewing the patients were defined as; nephrotoxicity (doubling of Cr from baseline), hypokalaemia (serum K+ < 3.4 mEq/L) and anaemia (haemoglobin drop 5 g from baseline) [10]. In the event of severe symptomatic anaemia, the patient was referred to hospital for blood transfusion. The protocol recommended the start of anti-retroviral treatment within 4–6 weeks of starting cryptococcal meningitis treatment if the patient showed clinical improvement. Retrospective application of the Division of AIDS (DAIDS) classification was also carried out for comparison with other studies [11].

Data were anonymised and entered directly onto an Excel spreadsheet by two investigators (CW, CM). Once complete, the database was locked and data analysed using Stata version 13. This project was a service evaluation, therefore it was deemed exempt from needing ethics reviewin accordance with Oxford Tropical Research Ethics Committee guidelines.

## Results

### Demographic factors

In total, 76 HIV positive patients diagnosed with cryptococcal meningitis were treated at MAM clinics from January 2010 to December 2017 (see Table 1). Forty-eight patients were male (63%), with a median age of 35 (lower, upper quartiles 30, 38) years at diagnosis (range 14–63 years). Mean body mass index was 18.2 kg/m^2^ (range 12–26 kg/m^2^).

**Table 1:** Baseline characteristics of patient with Cryptococcal Meningitis Diagnosis between 2010 and 2017

Characteristic	Variable	Total (***N*** = 76)^**a**^
Demographics	Age	35 (30, 38)
	Male gender	48/76 (63%)
History	Concurrent tuberculosis treatment	33/74 (45%)
	Concurrent OI (e.g. oesophageal candidiasis, MAC)	27/76 (36%)
	IRIS	25/75 (33%)
Symptoms	Headache	47/64 (73%)
	Fever	33/59 (56%)
	Weight loss	31/60 (52%)
	Altered mental state	58/76 (76%)
Signs	Focal neurology	12/51 (24%)
	Skin lesions	15/20 (75%)
	Abnormal chest examination	5/63 (7.9%)
Investigations	CD4 count- at time of CM	60.0 (31.0, 112.0)
	Serum CrAG positive	33/39 (85%)
	Split skin smear positive	16/31 (52%)
	CSF Opening pressure > 20 cm	35/72 (54%)
	CSF India Ink positive	50/70 (73%)
	CSF CrAG positive	16/23 (70%)

*Abbreviations*: *CM* cryptococcal meningitis, *CrAG* cryptococcal antigen, *CSF* cerebrospinal fluid, *OI* opportunistic infection, *IRIS* immune reconstitution inflammatory syndrome, *MAC Mycobacterium avium* complex

^a^n/N (%) or median (25^th^, 75^th^ percentile)

### Cryptococcal meningitis

At the time of cryptococcal meningitis diagnosis, the median CD4 count was 60 (lower, upper quartiles 31, 112). Overall the median duration of amphotericin-B-deoxycholate delivery was 14 days (8.5, 14 ). Forty-three patients had repeat LPs as part of their investigation and management. The average number of LPs per patient was 3 (range 2–17). Fourteen patients started ART within the recommended 4–6 weeks post diagnosis. Immune reconstitution inflammatory syndrome (IRIS) within 6 months of starting HIV treatment was identified in 25/75 (33%) of cases. Concurrent tuberculosis treatment was being delivered in 33/74 cases (45%), and 27/76 (36%) were diagnosed with at least one other WHO recognised opportunistic infection. The commonest presenting symptom was headache 47/64 (73%). Complaints of fever 33/59 (56%) and weight loss 31/60 (52%) were reported less frequently.

Serum CrAg testing was introduced routinely in 2011, this was positive in 33/39 (85%) of cases. Split skin smears were positive in 16/31 (52%) of cases. Cerebrospinal fluid analysis was carried out on 72 patients, an opening pressure > 20 cm H_2_0 (range 20–80 cm H_2_0) was present in 35/72 (50%), india ink was positive in 50/70 (73%), CSF CrAg was performed on 24 occasions, 16/23 (70%) were positive (see Table 1).

### Ambulatory care outcomes

**Fig. 1:**
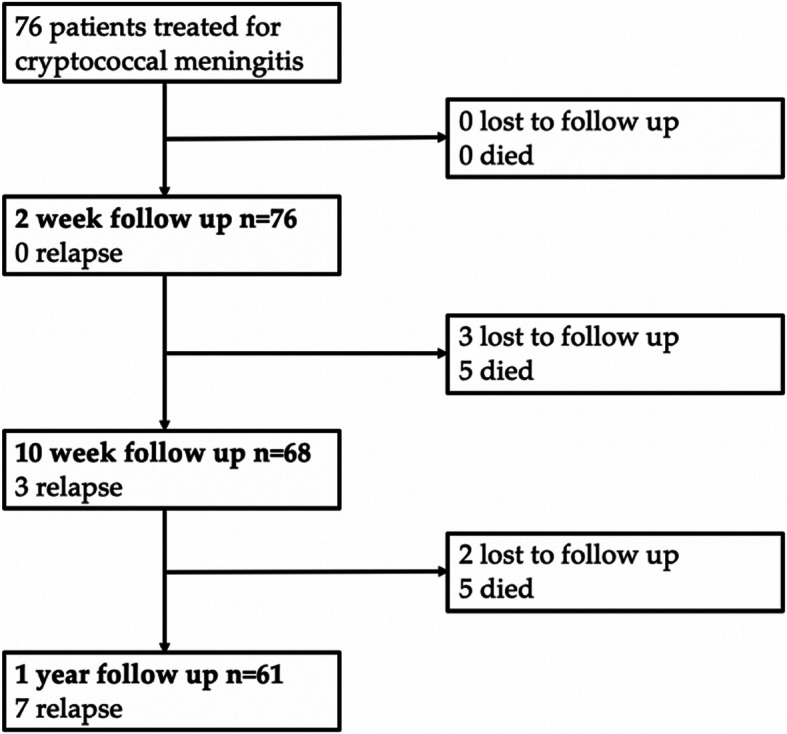
Patient outcomes on the ambulatory treatment pathway

There were 76 patients at baseline, at one year 5 patients had been lost to follow up, and mortality was 10/71 (14%). Overall, 19/76 (25%) patients were transferred to hospital due to deterioration; in this group at one-year post-baseline 2 patients had been lost to follow up and 5/17 (29%) patients died (see Fig. 1).

The tariff for a secondary referral hospital bed per day in the Yangon region is estimated at $22.11 [12]. Therefore, ambulation of patients on intravenous amphotericin-B-deoxycholate in this cohort saved 1029 hospital bed days ($22,751.19).

### Amphotericin toxicity

Amphotericin-B-deoxycholate infusions precipitated 56 episodes of ‘toxicity’ in accordance with the Médecins Sans Frontières 2015 guidelines, [10] observed in 44 patients (58%). These complication episodes (for definitions see methods) included; nephrotoxicity 24 (43%), hypokalaemia 21 (38%), phlebitis 5 (9%), febrile reaction 4 (7%) and anaemia 2 (4%). No patient died during the 2 weeks of amphotericin treatment. Despite these drug complications among 44 patients, 24 individuals (32 toxicity events-12 nephrotoxicity, 3 phlebitis, 13 hypokalaemia, 3 febrile reactions, 1 anaemia) went on to complete 14 days of amphotericin. See Table 2.

**Table 2:**
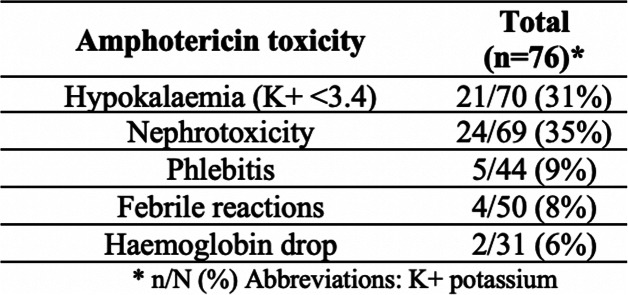
Amphotericin toxicity

*n/N (%) Abbreviations: K + potassium

If the DAIDS classification system were applied to these data instead the following can be deduced. Grade 3 anaemia (hemoglobin [Hb] < 7.5 g/dL) was observed in 6 cases (19% of those tested), and Grade 4 anaemia (Hb < 6.5 g/dL) was observed in 7 cases (22% of those tested). Grade 3 creatinine rises (increase to 1.5 to < 2.0 x participant’s baseline) were observed in 17/69 cases (24% of those tested), and Grade 4 creatinine rises (increase of ≥2 x baseline) were observed in 23/69 cases (33%). Grade 3 hypokalaemia (2.0- < 2.5 mEq/L) was observed in 6/70 patients (9% of those tested), Grade 2 hypokalaemia (2.5- < 3.0 mEq/L was observed in 8/70 patients (11% of those tested.)

### Cryptococcal Meningits complications

Recurrence of cryptococcal meningitis occurred in 10 patients (13%); three had not completed the induction phase due to complications. Unmasking IRIS, within 6 months of anti-retroviral therapy initiation occurred in 25 cases (33%) 7–149 days post initiation of antiretroviral treatment (median 45 days), and 4 patients had paradoxical IRIS after staring ART.

## Discussion

This case note review offers insight into demographics of HIV positive cryptococcal meningitis patients and their management outcomes from ambulatory treatment in specialist outpatient clinics in Yangon, Myanmar. This report highlights that ambulatory management of cryptococcal meningitis is possible in resource constrained settings. It reveals the provision of accessible, life-saving treatment among a stigmatized population of patients living with HIV. Ambulatory care avoids admission to hospitals in which services are limited and expensive in this setting.

Based upon existing estimates that cryptococcal meningitis prevalence is 6% in patients with CD4 < 100 at the time of antiretroviral initiation, [9] we believe the 76 patients included in this study is representative of the cases with CD4 < 100 (1460 patients) within the MAM cohort during this study period. The outcomes described suggest that mortality within the first 2 and 10 weeks of treatment (0 and 7%) were much lower than comparable published data on LMIC hospital-based cohorts in (~ 17% at two weeks and 34% at 10 weeks [13, 14]). The relevant population in this study are patients presenting to a community outpatient clinic, which you would expect would have lower mortality than previous studies where the population are hospitalised patients. Acutely unwell patients may have presented directly to hospital in preference to a community outpatient clinic, or succumbed prior to presenting to a health center. Existing literature is limited to hospital-based treatment, and our ambulatory pathway demonstrates clinical experience of reduced bed blocking, which is a principal aim of current randomised trials.

Amphotericin-B-deoxycholate toxicity definitions were based on the treatment guidelines that the clinicians were using at the time of assessment, to review adjustment of therapy in response to results and subsequently the patient outcome. DAIDS were also used retrospectively showing increased toxicity levels, particularly nephrotoxicity compared to other published hospitalised cohorts [5–7] Off site biochemistry testing prevented same day response to derangement of potassium or creatinine. Despite this, the outcomes of patients whose treatment continued despite toxicity was much better than might otherwise have been predicted. Toxicity events observed highlight the complications of ‘old’ drugs, and serves as a reminder that access to affordable flucytosine and liposomal amphotericin-B is still not achievable in resource constrained settings. Further lobbying of drug companies, bilateral organization and governments is needed to facilitate provision of cost-effective treatments in areas that need them most.

We welcome the increasing evidence supporting reduced duration of induction phase toxic treatment. The Advancing Cryptococcal meningitis Treatment for Africa (ACTA) trial demonstrated seven-day courses of amphotericin-B-deoxycholate and flucytosine had lower mortality at 10 weeks (24%) compared to patients enduring 14-day induction therapy (38% mortality). ACTA also demonstrated superiority of flucytosine over fluconazole treatment [15]. Follow up studies supported this approach [16], and the WHO guidelines were revised in response to their findings. The AMBIsome Therapy Induction OptimisatioN (AMBITION) study [17] is a phase 3 trial (recently completed recruitment at time of publication) that will provide important insight into whether single high dose liposomal amphotericin with 14-days high dose flucytosine and fluconazole therapy is non-inferior to the WHO recommended 7-day course of amphotericin-B-deoxycholate and flucytosine, followed by 7 days fluconazole in cryptococcal meningitis cases. Single high dose amphotericin treatment could revolutionise management and enable a more widespread ambulatory approach to care. However, both the ACTA and potential AMBITION trial findings rely on flucytosine becoming affordable and accessible globally to become translatable to the international setting such as MAM clinics.

Limitations of this analysis include, lost patient files, changes in laboratory diagnostic investigations (e.g. cryptococcal antigen not introduced until 2011) and treatment guideline revisions during this retrospective review restricting detailed analysis of outcomes. The number of patients was too small to accurately identify predictors of death. Documentation of fluconazole dosing and side-effects was too incomplete for analysis.

Gold standard CSF fungal culture was not available. Therefore, extrapolation of these results and comparison with established studies with more robust diagnostics is not possible. Delineation of the precise cause for recurrent infection was compromised by these laboratory limitations. However, we believe cryptococcal meningitis was a secure diagnosis in this cohort, with 73% CSF samples India Ink positive and 85% CRAG positive. The findings of this service review were used to internally audit the diagnosis and management of cryptococcal meningitis in our clinics to ensure the highest standards of patient care in this setting are delivered and maintained.

## Conclusions

Our study population represents the large (but poorly-addressed) majority of HIV immunosuppressed people in resource-constrained settings where high-tech diagnostics and unaffordable flucytosine recommended by WHO guidelines are not currently attainable for the management of cryptococcal meningitis [3]. This paper outlines an economical ambulatory approach to HIV associated cryptococcal meningitis treatment using amphotericin-B-deoxycholate and fluconazole. Prompt response to blood results, especially potassium levels is recommended. We view this ambulatory pathway as an acceptable alternative to the unachievable regimens recommended in international guidelines. Outpatient treatment with intravenous amphotericin-B-deoxycholate and oral fluconazole is a relatively inexpensive, practicable and acceptably effective option to be considered in resource constrained settings.

## Data Availability

The data that support the findings of this study are available from the corresponding author upon reasonable request and with permission of Myanmar Oxford Clinical Research Unit.
